# Silicate Mineral Eutectics with Special Reference to Lithium

**DOI:** 10.3390/ma14154334

**Published:** 2021-08-03

**Authors:** Agata Stempkowska

**Affiliations:** Department of Environmental Engineering, Faculty of Civil Engineering and Resource Management, AGH University of Science and Technology, Mickiewicza 30 Av., 30-059 Cracow, Poland; stemp@agh.edu.pl

**Keywords:** Li-Na-K flux system, mineral eutectic, spodumene

## Abstract

In this paper, the system of natural mineral alkali fluxes used in typical mineral industry technologies was analyzed. The main objective was to reduce the melting temperature of the flux systems. Particular attention was paid to the properties of lithium aluminium silicates in terms of simplifying and accelerating the heat treatment process. In this area, an alkaline flux system involving lithium was analyzed. A basic flux system based on sodium potassium lithium aluminosilicates was analyzed; using naturally occurring raw materials such as spodumene, albite and orthoclase, an attempt was made to obtain the eutectic with the lowest melting point. Studies have shown that there are two eutectics in these systems, with about 30% spodumene content. The active influence of sodium feldspar was found.

## 1. Introduction

### 1.1. Characteristics of Flux Minerals

Lithium is a very common yet dispersed element. The largest resources are found in seawater—billions of tons of highly diluted lithium (the range is from 0.001 to 0.020 mg/L lithium), which cannot be extracted on an industrial scale [1,2]. In dissolved form, lithium has only positive effects on supplementation of its deficiencies in humans [3,4]. Minerals containing lithium in nature are formed from the transformation of pegmatites. Although about 145 minerals contain this element in smaller or larger amounts, the basic industrial raw materials are spodumene, lepidolite, petalite and amblygonite [5,6]. In industry, mainly lithium-containing aluminium silicates and sometimes lithium carbonate, obtained from lithium salines or from other lithium minerals mainly spodumene, are used. The use of lithium in the form of carbonate for high-temperature processes (firing) can cause a problem with the outgassing of the resulting CO_2_ [7,8,9]. For this reason, this paper is limited to the characterization of natural varieties of lithium aluminum silicates—spodumene. 

Spodumene LiAlSi_2_O_6_ is one of three natural varieties of lithium aluminum silicate, the others being petalite and eucryptite. However, eucryptite occurs in nature only in one deposit Bikita, Southern Rhodesia. Eucryptite is usually synthesized and available in the β variety [10,11]. Murthy and Hummel analyzed the phase system of lithium metasilicate and β-eucryptite. They found that the eutectic is formed at about 1070 °C [12,13]. Spodumene is a mineral of the pyroxene group. It crystallizes in a single-crystal system in the space group C2/c forming sometimes quite large crystals of whitish, gray, yellowish, lilac (kunzite) or emerald green (hiddenite) color. Spodumene melts at a temperature of about 1420 °C. However, in combination with quartz, feldspars, also mica, it forms low-temperature eutectics [14,15] At room temperature spodumene occurs in the α form. This form has a density of 3.2 g/cm^3^. At a temperature of about 1080 °C, an irreversible transition of the α form to β occurs. This involves a rearrangement of the structure from a single chain to a less dense tetragonal structure. The density of β-spodumene is 2.4 g/cm^3^. The change in density represents a 30% increase in volume [6,16].

The formation of a vitreous phase in a mineral mass requires the addition of fluxes. One of these is spodumene. With spodumene, it conducts the firing so that the following reaction occurs [17]:

Li_2_O-Al_2_O_3_-4SiO_2_ + 4SiO_2_ → Li_2_O-Al_2_O_3_-8SiO_2_ (1)

spodumene    silica    β-spodumene (solid solution)

The next minerals in the studied flux systems are alkali feldspars, these are raw materials rich in potassium (K_2_O), which are bound in them mainly in the form of aluminosilicates, i.e., potassium feldspars (orthoclase, microcline, sanidine, adular). For this reason, anorthite, Ca[Al_2_Si_2_O_8_], whose melting point is high (1550 °C) should not be included in these raw materials although small amounts can produce eutectic. The melting point of alkali feldspars is significantly lower [18,19].

The other feldspar used as a flux is albite. Albite in nature never occurs in the pure form of sodium aluminosilicate and always contains some amount of calcium. An exception is a raw material under the name Albitte 5 (Turkey), which contains less than 1% calcium oxide and can be treated as a pure mineral [20,21].

Stechiometric albite contains 11.8 wt.% Na_2_O, 19.4 wt.% A1_2_O_3_ and 68.8 wt.% SiO_2_. Its melting point is 1120–1200 °C and its density is 2.62 g/cm^3^. The characteristic of albite is lower melt viscosity compared to other feldspars. In general, it is a better flux than potassium feldspar because it melts at a lower temperature; however, it causes greater deformation in the material because of congruent melting [22].

Potassium feldspar (orthoclase) K[AlSi_3_O_8_] melts incongruently, while sodium feldspar (albite) Na[AlSi_3_O_8_] melts congruently. During the firing process, the silicate melt formed at the expense of the feldspar interacts with the solid phase and partially dissolves it. This phenomenon starts at a temperature of about 1150 °C. The silicate alloy leads to the expected thickening and lower porosity of the ceramic material. Feldspar raw materials characterized by the predominance of K_2_O to Na_2_O (K_2_O/Na_2_O ratio should be more than 2) are most often used in technologies. These criteria are based on theoretical premises, while there is no sufficient research data in this direction, although in the case of mineral materials it is justified by the incongruent melting of potassium feldspar [22,23].

Due to the economic and environmental aspects (fossil fuel consumption, greenhouse gas emissions), research work on lowering the temperature and reducing the firing and melting time of mineral products has been going on for many years [24,25].

### 1.2. Selected Thermal Parameters of Mineral Flux Systems

The energy absorbed by a body during its heating or given off during its cooling is proportional to the product of the body mass m and the temperature difference of this body ΔT before and after the thermal transformation. The thermal transformation ability of a material ΔQ can be written in the form:

ΔQ = c_v_·m·ΔT (J) (2)

The heat capacity of a material is called the amount of heat required to raise the temperature of a substance by one degree. The heat capacity that accrues per unit mass of a substance is called the specific heat c_v_ (expressed in J/kg·K). This quantity is not a constant value and depends primarily on temperature. The specific heat is an additive quantity, i.e., each degree of freedom present in a system contributes to the total heat of the system. In many amorphous, glassy and crystalline substances, the specific heat increases with increasing temperature and at high temperatures [26,27,28].

Another quantity that characterizes materials in terms of their thermal properties is the volumetric heat capacity. Its value b, is calculated as the product of the specific heat, c_v_ and the material density, ρ, of which the material is made:

b = c_v_·ρ (J/(m^3^K)) (3)

Volumetric heat capacity is a measure of the amount of energy that 1 m^3^ of a given material will absorb while heating or give up while cooling, changing its temperature by one degree. In other words, it is the energy that raises (or lowers) the temperature of a material of unit volume by unit temperature. The materials with the highest specific density have the highest heat capacity. However, volumetric heat capacity is not sufficient to describe the ability to accumulate heat. The energy that can be accumulated per unit volume of a material is an additional parameter characterizing the effectiveness of the material accumulation phenomenon. The maximum energy that can be accumulated in a unit volume of a given material b_maxv_ can be described by the formula [29]:

b_maxv_ = b·Δt (J/m^3^) (4)

The values of maximum volumetric energy for different rock types are in the range of 0.5–3.5 kJ/m^3^ [30,31]. The eutectic transformation during heating requires a certain heat power P, which is given by the relation [32]:

P = ∆Q/t (J/s)(5)

where: ΔQ—thermal transformation, t—heating time.

The main objective of this study was to determine the image of phase transformations temperature-eutectics. The second objective was to calculate and visualize the thermal parameters of the thermal transformations of the three-component system.

## 2. Materials and Methods

In contrast to the synthetic components of the flux system, a characterization is presented for the determination of fluxing eutectics based on naturally occurring alkalines (Table 1) [33]. For naturally occurring lithium aluminosilicate minerals, temperatures can vary significantly. Therefore, it is advisable in each case to determine the eutectic points of mixtures of sodium potassium feldspar and naturally occurring lithium aluminosilicates for use as fluxes.

Naturally occurring and commercially available raw materials containing alkali minerals were used to achieve this goal. For lithium aluminium silicate, Gresflux and micronized Concentrate were used. For Na and K feldspar, Albitte 5 nd Norfloat Spar were used. All natural mineral raw materials were supplied by Otavi minerals (Neuss, Germany). The compositions of each raw material in terms of oxides are shown in Table 2.

**Figure 1:**
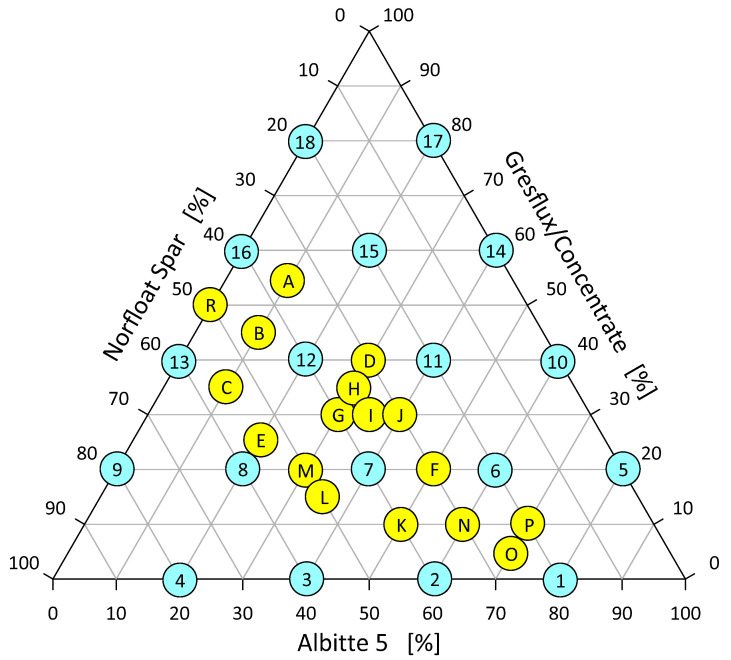
Distribution of samples in the spodumene–albite–microcline triangle.

Base sets differing by 20% of the individual components were prepared, then the composition of the sets was condensed into the eutectic region differing by 5 wt.% and 10 wt.%. as shown in Figure 1, tests were performed for both Gresfux spodumene and Concentrate.

**Figure 2:**
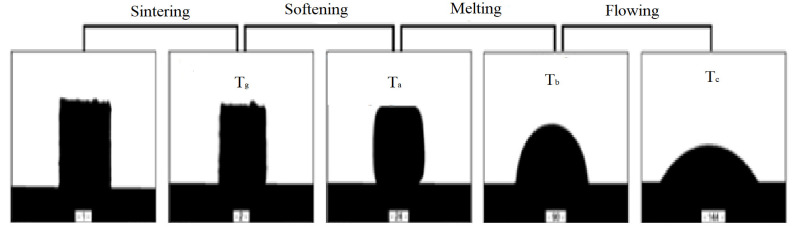
Method of determining characteristic temperatures and intervals in high-temperature microscopy, original state of sample 1 (spodumene Gresflux).

To determine the effect of enrichment and grinding of the lithium component, enriched and micronized spodumene Concentrate with an average grain size of d_50_ = 3 µm and ground spodumene Gresflux with a starting grain size of d_50_ = 180 µm were introduced into the sets. Regardless of the degree of grinding, all sets were additionally homogenized for about 15 min in an alumina mill Fritsch pulverisette MV46 (Idar-Oberstein, Germany). The average grain size distribution of the sets did not exceed 50 µm. Measurements in high-temperature microscope Hesse-Instruments EM301 (Osterode am Harz, Germany) were performed on particular sets of specimens with the following assumptions; temperature increment from 80 °C/min to 650 °C and 10 °C/min in the temperature range from 650 °C to 1500 °C. On the basis of continuous observation of the specimen and recording changes in its dimensions as a function of temperature, it is possible to determine the so-called characteristic temperatures (Figure 2) [14,15].

− shrinkage starting temperature T_g_ (sintering)
− softening temperature T_a_ (corner rounding-end of sintering)
− melting point T_b_ (hemispherical effect-formation of mineral eutectic)
− spreading temperature T_c_ (sample base >200% or 1/3 height)

By computer recording the change in the shape of the samples, it is also possible to determine:

− sintering interval (corner rounding temp. T_a_-shrinkage temp. T_g_)
− melting interval (hemisphere temperature T_b_-corner rounding temperature T_a_)
− flowing interval (spreading temp. T_c_-hemisphere temp. T_b_),

Investigations carried out in the high-temperature microscope belong to the standard investigations of thermal properties of materials. Not only do they allow the determination of characteristic temperatures, but also decomposition temperatures, sublimation temperatures, phase transition temperatures, etc. On the basis of continuous observation of the sample and recording changes in its dimensions as a function of temperature, it is also possible to determine the viscosity of the alloy or wetting ability with respect to the substrate [34]. Data visualization was performed with the Surfer 19 program from GoldenSoftware delivered by Gambit (Kraków, Poland). The presented test results are the average values from three measurements.

## 3. Results and Discussion

### 3.1. Characteristic Temperatures

**Figure 3:**
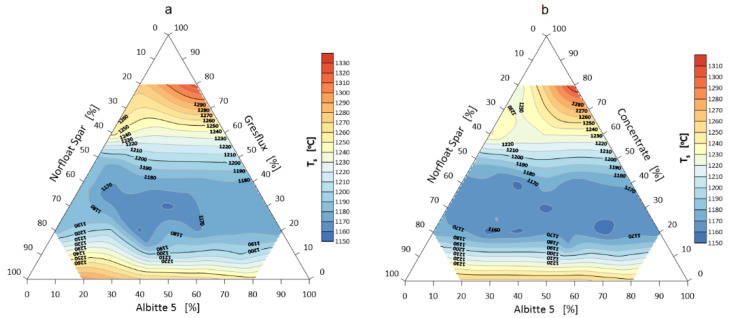
Shrinkage onset temperature T_g_ of the flux system: Greflux–Albitte 5–Norfloat Spar (**a**), and Concentrate–Albitte 5–Norfloat (**b**).

**Figure 4:**
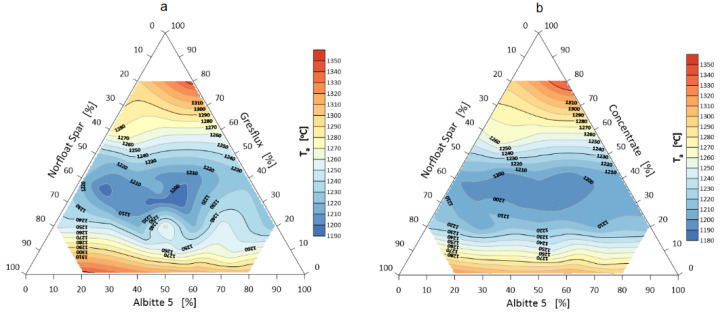
Rounding temperature T_a_ of the corners of the flux system Greflux–Albitte 5–Norfloat Spar (**a**), and Concentrate–Albitte 5–Norfloat Spar (**b**).

The onset of shrinkage of individual sets during heating is the beginning of sintering and the extent to which the corners round off defines the extent of sintering. The volume change during this phenomenon is probably determined by three mechanisms. The first is related to the thermal expansion of the grains, which induces an approximate 1.5% volume swelling of the samples up to a temperature of about 1000 °C. In the case of spodumene, the second swelling mechanism (about 30 vol.%) in the temperature range 1050–1100 °C is related to the polymorphic transformation of α to β spodumene [35,36]. In the next heating stage, shrinkage occurs due to sintering, melting and elimination of the gas phase. The temperature of the beginning of softening, i.e., rounding of corners (end of sintering) lies above the temperature of polymorphic transformation in which a significant volume change occurs. The temperature of onset of shrinkage and rounding of corners are shown in Figure 3 and Figure 4.

**Figure 5:**
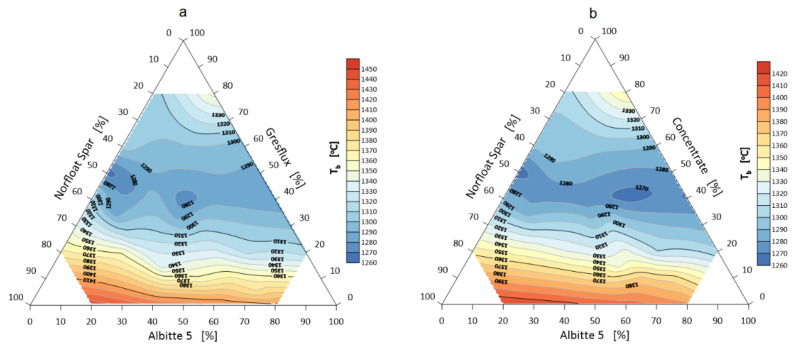
Melting temperature (hemisphere) T_b_ of the flux system Greflux–Albitte 5–Norfloat Spar (**a**), and Concentrate–Albitte 5–Norfloat Spar (**b**).

The stage of eutectic formation of the studied system, in general, was defined as the change in the shape of the samples from the temperature of rounding of the corners, i.e., the beginning of their softening, to the formation of a hemisphere (Figure 2). The visualization of these measurements is shown in Figure 5.

**Figure 6:**
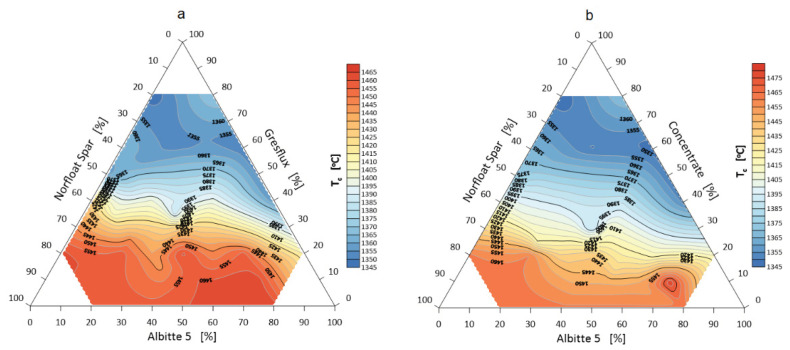
The beginning of the flux system spreading T_c_, Greflux–Albitte 5–Norfloat Spar (**a**), and Concentrate–Albitte 5–Norfloat Spar (**b**).

The measurements showed the first eutectic point, i.e., 1263 °C (Concentrate) and 1273 °C (Gresflux) at compositions of 50 wt.% potassium feldspar and 45 wt.% spodumene and 30 wt.% potassium feldspar, 30 wt.% sodium feldspar and 40 wt.% spodumene, respectively. This applies to both micronized (d_50_ = 3 µm) and ground spodumene (d_50_-180 µm). A second eutectic with a higher melting point of 1382 °C (Gresflux) and 1376 °C (Concentrate) exists at the point with proportions of 50/45/5 wt.% (B-eutectic). The study showed that the melting point of the different sets, measured as the point of hemisphere formation, did not differ significantly depending on the fineness and enrichment of the spodumene. Using micronized spodumene (Concentrate), the melting point of the system can be lowered, at most, by about 10 °C relative to ground spodumene (Gresflux). This fact can probably be explained by the influence of the spodumene surface development and the increased amount of lithium by about 1.4 wt.% (Table 2) compared to the Gresflux spodumene. An active effect of sodium feldspar on the melting point of the system can be observed as well as a shift of the eutectic towards higher spodumene contents. The study shows that spodumene decreases the melting temperature of the system more than other feldspars. The characteristic spreading temperatures are shown in Figure 6.

**Figure 7:**
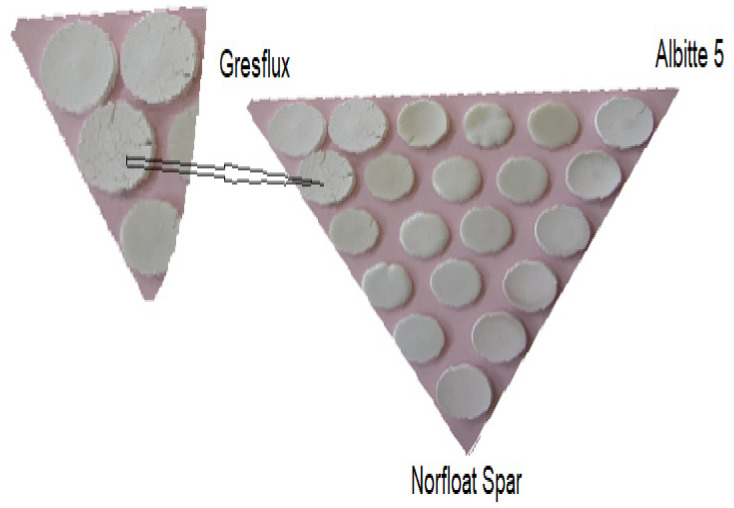
Swelling phenomenon of flux systems containing increased amounts of spodumene, (diameter of the pastille was 5 cm).

It can also be observed that spodumene (both Gresflux and Concentrate) in amounts above 60 wt.% causes significant swelling of samples up to 35 vol.% (Figure 7).

In the case of mineral materials, high swelling can lead to the deformation of the products. In the case of an alloy, there is no risk and the so-called softening stage is usually defined as the difference between the temperature of the onset of shrinkage and the temperature of the hemisphere. In the case of alloys, this is justified by the usually narrow transition interval between solid and liquid. From the functional point of view in terms of meltability, the reactivity of spodumene at small amounts in the set, up to 5 wt.%, is interesting. This fact is not known so far in the literature.

### 3.2. Sintering, Melting and Flowing Intervals

**Figure 8:**
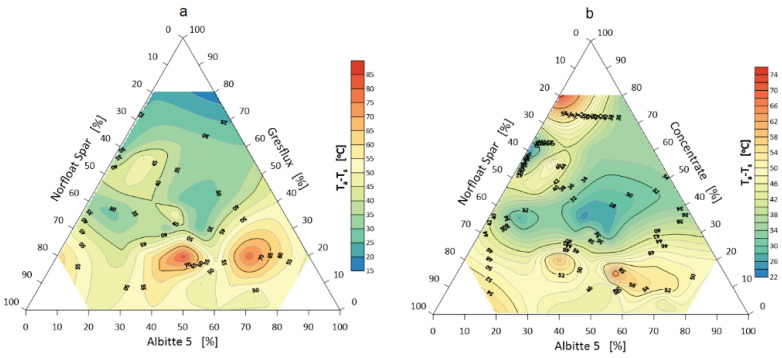
Sintering interval of the flux system Greflux–Albitte 5–Norfloat Spar (**a**), and Concentrate– Albitte 5–Norfloat Spar (**b**).

The sintering interval of the sets, calculated from the temperature of the beginning of contraction to the temperature of the rounding of the corners, is of less importance with respect to flux systems. However, the smaller the interval is, the faster the system goes to the melting phase (faster time of eutectic transformation). Studies have shown that the sintering interval is shorter the more the composition of the sets approaches the eutectic point. One can also notice a greater discrepancy in the measurement results when Gresflux is used especially at a higher proportion. Probably the swelling effect caused by the polymorphic change of spodumene has a significant influence in this case. It is worth noting the two increased sintering intervals at 20 wt.% spodumene and 40 and 70 wt.% albite, respectively (Figure 8).

**Figure 9:**
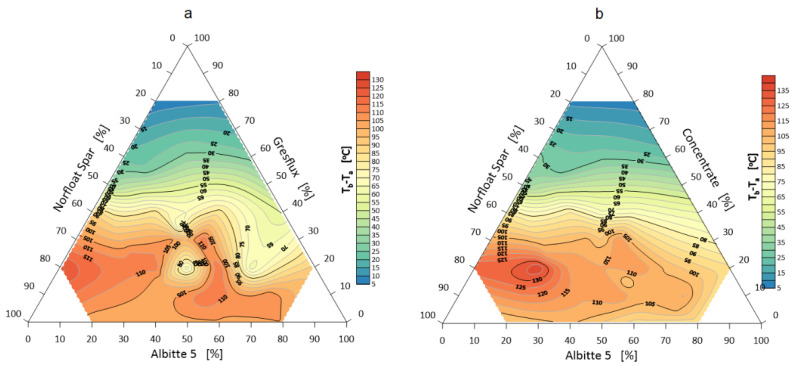
Melting interval of flux system Greflux–Albitte 5–Norfloat Spar (**a**), and Concentrate–Albitte 5–Norfloat Spar (**b**).

Melting of the system begins when the corners of the samples are rounded. The melting interval measured by the difference between the corner rounding temperature (end of specimen shrinkage and corner rounding) and the melting temperature (hemisphere) is shown in Figure 9.

**Figure 10:**
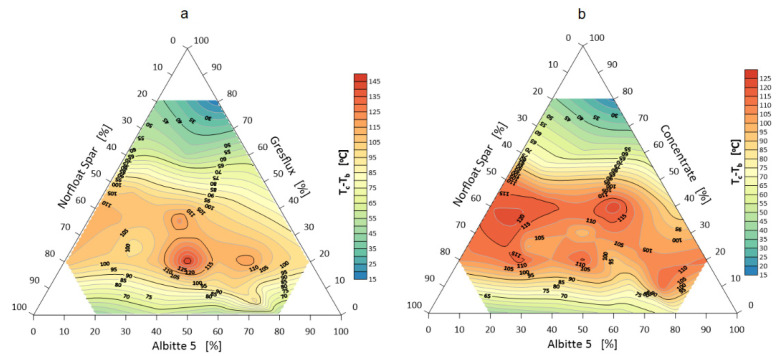
Flowing interval of a flux system Greflux–Albitte 5–Norfloat Spar (**a**), and Concentrate–Albitte 5–Norfloat Spar (**b**).

The total softening interval of the system increases slightly in favor of additional milling and enrichment. This phenomenon is related to the surface development of the micronized lithium aluminum silicate and faster reaction in the melting range. The flowing interval determines the difference between the spreading temperature and the melting temperature (hemisphere temperature). The results are visualized in Figure 10.

Again, the greater fineness and enrichment of spodumene reduces the melt flow interval of the system by about 10 °C. The effect is particularly pronounced with increased spodumene content. Technological experience indicates that a smaller melt flow interval (lower melt viscosity) is more advantageous for the preparation of vitreous coatings as it allows better gas-phase evacuation and thus a reduction in the number of defects. In this respect, for the production of amorphous coatings, a larger spodumene grain size is not advantageous.

### 3.3. Thermal Parameters of a Three-Component Flux System

On the basis of the percentage of particular oxides contained in the raw materials (Table 2) and their tabulated c_v_ values (Table 3), the resultant specific heat of the materials studied was calculated. The highest value of resultant specific heat is characterized by spodumene micronized Concentrate, it is 828.95 J/kgK, (Table 4). Specific heat is a quantity that is particularly sensitive to phase transformations of all types, i.e., a quantity whose value changes significantly near phase transformations. It carries valuable information about the nature, latent heat or critical exponent of the transformation. The incidental specific heat depends closely on the chemical composition of the raw material, which is why quality control is so extremely important. In the calculations, reference was made to the oxides of the individual elements, which build natural minerals, for the reason that the spatial structure of silicates consists of tetrahedral metal–oxygen bonding [22].

The materials with the highest specific density are characterized by the highest thermal storage capacity b. Volumetric heat capacity of metals with density 7000–9000 kg/m^3^ is 1.5–3.5 MJ/(m^3^ K). Natural rock formations have lower volumetric heat capacity than metals, e.g., granite—about 1.8 MJ/(m^3^ K) gabbro about 2.2 MJ/(m^3^ K), granodiorite about 2.3 MJ/(m^3^ K). Even smaller volumetric heat capacity is characterized by brick and sand—about 1.2 MJ/(m^3^ K) [29,31,37]. Mineral eutectics are characterized by similar values of parameter b to natural rocks. Analyzing the thermal accumulation capacity b contained in Table 5 and Table 6, it was found that the highest value is characterized by sample 18 with a composition of 20 wt.% albite and 80 wt.% spodumene, and this applies to both types of spodumene Gresflux and Concentrate. This seems to be due to the synergistic effect that sodium and lithium have on each other [14].

**Figure 11:**
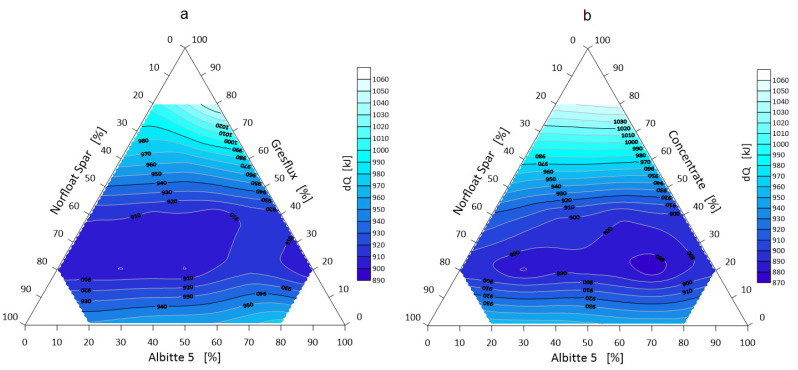
Transformation energy ∆*Q* of the three-component system, Greslux (**a**)/Concentrate (**b**) –Albitte 5–Norfloat Spar.

**Figure 12:**
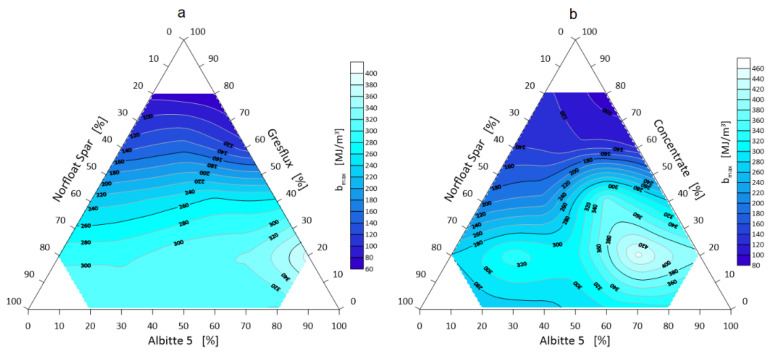
Maximum volumetric energy bmax of the three-component system Greslux (**a**) /Concentrate (**b**) –Albitte 5–Norfloat Spar.

**Figure 13:**
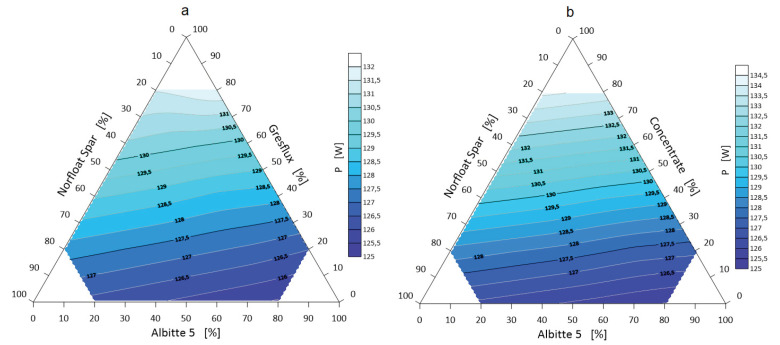
Thermal power P of the Greslux (**a**)/Concentrate (**b**) –Albitte 5–Norfloat Spar three-component mineral system.

The next step was to visualize selected thermal parameters of the spodumene–albite–microcline (Li-Na-K) three-component system. These data are presented in Figure 11, Figure 12 and Figure 13.

The transformation energy ∆Q of a three-component mixture depends on the mass m, the resultant specific heat c_v_, and the temperature difference during thermal exposure. The temperature difference is the beginning of heating of the system and reaching its melting temperature, that is phase transformation (hemisphere point of Figure 2). From a technological point of view, the lowest energy value is the most desirable—a mineral melt is obtained with the minimum possible heat input. Figure 11 shows the thermal transformation ∆Q map of the system. It can be seen the fields with the lowest values at a spodumene content of about 30 wt.%. When using micronized spodumene, the minimum energy value is about 20 kJ lower (Gresflux, lowest energy fields 890 kJ, while Concentrate 870 kJ).

The maximum thermal energy that the system is able to accumulate depends directly proportional to the density of the components, and to the calculated specific heat (Table 4). The highest value of the maximum volumetric energy amounting to over 440 MJ/m^3^ exactly (447 MJ/m^3^) was obtained in the system with a component proportion of 20 wt.% micronized spodumene, 20 wt.% microcline and 60 wt.% albite (point 6 in Figure 1), where the effect of grain size is clearly visible (Figure 12).

The heat power of the mineral set is linear and increasing with the spodumene content. The thermal power strictly depends on the amount of energy consumed by the system ∆Q in a given time unit. The time of energy accumulation *t* was taken as the moment of eutectic formation (Table 5), and the whole system was heated at 10 °C/min in the temperature range 650–1500 °C. For the micronized Concentrate spodumene, the ∆Q values were several watts higher. This is due to the higher calculated specific heat (Table 4).

## 4. Conclusions

The degree of milling of spodumene concentrate lowers the eutectic temperatures of the system of spodumene–albite–microcline, but this change is marginal. In the case of the characteristic hemisphere temperature, the change is about 12 °C and the softening temperature (rounding of corners) is about 10 °C.

It was observed that micronized spodumene concrete undergoes a faster polymorphic transformation. This phenomenon causes less swelling and thus prevents the cracking of the samples at spodumene contents >60%. Furthermore, a significant effect of spodumene grain size on all thermal parameters of the three-component system was observed.

The characteristic temperatures of the applied natural flux raw materials are different from those of pure synthetically derived minerals (literature data). In particular, the characteristic melting temperature (hemisphere) of spodumene as a synthetic component is lower by about 7–15 °C, while albite and microcline are lower by about 242 °C and 184 °C, respectively.

The first eutectic melting points of the mineral system spodumene (Gresflux/Concentrate)–potassium feldspar (Norfloat Spar)–sodium feldspar (Albitte 5) occurred at 1275 °C (Gresflux) and 1263 °C (Concentrate) with proportions of 40/30/30 wt.% (A-eutectic). A second eutectic with a higher melting point of 1382 °C (Gresflux) and 1376 °C (Concentrate) exists at the point with proportions of 50/45/5 wt.% (B-eutectic).

The fields of the lowest values of the energy of eutectic transformation are observed when the spodumene content (of both types) is about 30 wt.%. However, the maximum thermal energy accumulated by the system is 447 MJ/m^3^, which refers to the system with micronized spodumene with the composition of 20 wt.% Concentrate, 20 wt.% Norfloat and 60 wt.% Albitte 5.

Visualizations of selected thermal parameters of three-component mixtures clearly show that the formation of eutectic is the result of many parameters, however, strictly depends on the components’ proportions and their chemical composition.

The next stage of work will be the study of high-temperature viscosity of mineral eutectics, and the behavior of Li-Na-K system in the presence of raw materials containing Ca and Mg.

## Figures and Tables

**Table 1:** Characteristic temperatures of the synthetic components of the system.

**Melting Point (°C)**	**Spodumene**	**Albite**	**Microcline**
	1423	1118	1150

**Table 2:** Chemical composition and melting point of raw materials used.

Raw Material	Norfloat Spar	Albitte 5	Gresflux	Concentrate
Chemical Composition (wt.%)				
SiO_2_	65.9	67.1	68.0	64.95
Al_2_O_3_	18.5	18.8	22.0	26.80
CaO	0.5	0.6	0.4	0.05
MgO	0.1	1.3	0.2	0.00
TiO_2_	<0.05	0.36	-	-
Fe_2_O_3_	0.23	0.7	0.4	0.07
MnO	<0.05	<0.05	0.2	-
P_2_O_5_	0.06	0.13	0.3	0.12
**Na_2_O**	**2.9**	**9.5**	**1.0**	**0.15**
**K_2_O**	**12.0**	**0.2**	**1.0**	**0.08**
**Li_2_O**	**-**	**-**	**6.1**	**7.50**
LOI	1.02	1.49	0.29	0.23
Melting point (°C)	1434	1360	1414	1410
Dominant mineral	microcline	albite	spodumene	spodumene

**Table 3:** Tabular-specific heat.

Oxide	c_v_ (J/kg·K)
SiO_2_	742
Al_2_O_3_	775
Fe_2_O_3_	655
MgO	924
CaO	750
Na_2_O	1115
K_2_O	764
Li_2_O	1811

**Table 4:** Calculated specific heat and density of raw materials.

Raw Material	Norfloat Spar	Albitte 5	Gresflux	Concentrate
c_v_ (J/kg K)	762.63	772.14	811.79	828.95
density (g/cm^3^)	2.58	2.61	3.1	3.1

**Table 5:** Volumetric heat capacity b, and eutectic formation time, of three-component system: Gresflux spodumene, Albitte 5, Norfloat Spar, according to the compositions shown in Figure 1.

Set Number	b (MJ/(m^3^K))	t (s)	Set Number	b (MJ/(m^3^K))	t (s)
1	1.976	7794	A	2.268	7002
2	1.986	7656	B	2.213	7182
3	1.996	7620	C	2.159	6912
4	2.006	7542	D	2.196	6882
5	2.073	7098	E	2.111	6936
6	2.083	7224	F	2.092	6828
7	2.093	7074	G	2.141	6858
8	2.103	7056	H	2.169	6882
9	2.112	7044	I	2.143	6816
10	2.180	7146	J	2.147	6864
11	2.190	7044	K	2.042	7110
12	2.201	7050	L	2.067	6900
13	2.211	7062	M	2.093	6894
14	2.290	7560	N	2.049	7134
15	2.301	7410	O	2.027	7272
16	2.311	7428	P	2.054	7092
17	2.402	7650	R	2.223	7123
18	2.413	7998			

**Table 6:** Volumetric heat capacity b, and eutectic formation time, of three-component system: Concentrate spodumene, Albitte 5, Norfloat Spar, according to the compositions shown in Figure 1.

Set Number	b (MJ/(m^3^K))	t (s)	Set Number	b (MJ/(m^3^K))	t (s)
1	1.976	7578	A	2.295	7746
2	1.986	7566	B	2.235	7632
3	1.995	7602	C	2.175	7722
4	2.005	7596	D	2.215	7662
5	2.082	7044	E	2.121	7938
6	2.091	6858	F	2.102	7998
7	2.102	6936	G	2.156	7800
8	2.112	6864	H	2.185	7716
9	2.122	6942	I	2.158	7782
10	2.200	6954	J	2.161	7824
11	2.200	6882	K	2.047	8220
12	2.220	6942	L	2.074	8100
13	2.230	7056	M	2.102	7992
14	2.320	7410	N	2.053	8226
15	2.331	7422	O	2.029	8286
16	2.341	7392	P	2.058	8184
17	2.444	7902	R	2.255	8246
18	2.454	7830			

## Data Availability

The data presented in this study are available on request from the corresponding author.
